# Endoscopic ultrasound-guided antegrade stenting under guiding sheath assistance for malignant distal biliary obstruction

**DOI:** 10.1055/a-2573-8442

**Published:** 2025-05-12

**Authors:** Tadahisa Inoue, Rena Kitano, Tomoya Kitada, Kazumasa Sakamoto, Satoshi Kimoto, Jun Arai, Kiyoaki Ito

**Affiliations:** 1 12703 Department of Gastroenterology, Aichi Medical University, Nagakute, Japan

Endoscopic ultrasound-guided antegrade stenting (EUS-AGS) with/without hepaticogastrostomy (HGS) may achieve longer stent patency than EUS-HGS alone for malignant distal biliary obstruction (MDBO) with a transpapillary approach failure 1 2 . In addition, to retain the advantages of EUS-AGS while maintaining access routes and reducing bile leakage from the puncture site post-procedure, the utility of adding a plastic stent (PS) for HGS has also been reported 2 . This is primarily because EUS-AGS allows bile to flow physiologically and effectively into the duodenum, avoiding the peripheral bile duct obstruction that can occur with a metal stent (MS) for EUS-HGS. However, in EUS-AGS, guidewire passage through the stricture, which requires assistance with catheter insertion, is frequently demanding and time-consuming. In addition, intrabiliary pressure remains elevated until the antegrade stent is deployed, raising concerns about increased bile leakage, particularly during device exchange. To address these challenges, we propose a novel technique, EUS-AGS, with guiding sheath assistance.

A 65-year-old woman with pancreatic cancer and duodenal bulb stricture presented with obstructive jaundice secondary to MDBO. The left intrahepatic bile duct was punctured and a 0.025-inch guidewire was inserted into the common bile duct (CBD). Subsequently, a 7.2F guiding sheath 3 was advanced into the CBD and the inner catheter was removed. A 5.5F standard catheter then was inserted into the CBD through the outer catheter of the guiding sheath, which allowed the guidewire to advance across the stricture into the duodenum. Thereafter, a 5.4F-diameter MS delivery system was inserted through the guiding sheath with additional pushability support provided by the sheath and deployed antegrade across the stricture ( Fig. 1 , Fig. 2 , and Video 1 ). Finally, a 7F PS was placed in the HGS to maintain the access route after sheath removal. The patient's symptoms improved rapidly with no adverse events.

**Fig. 1:**
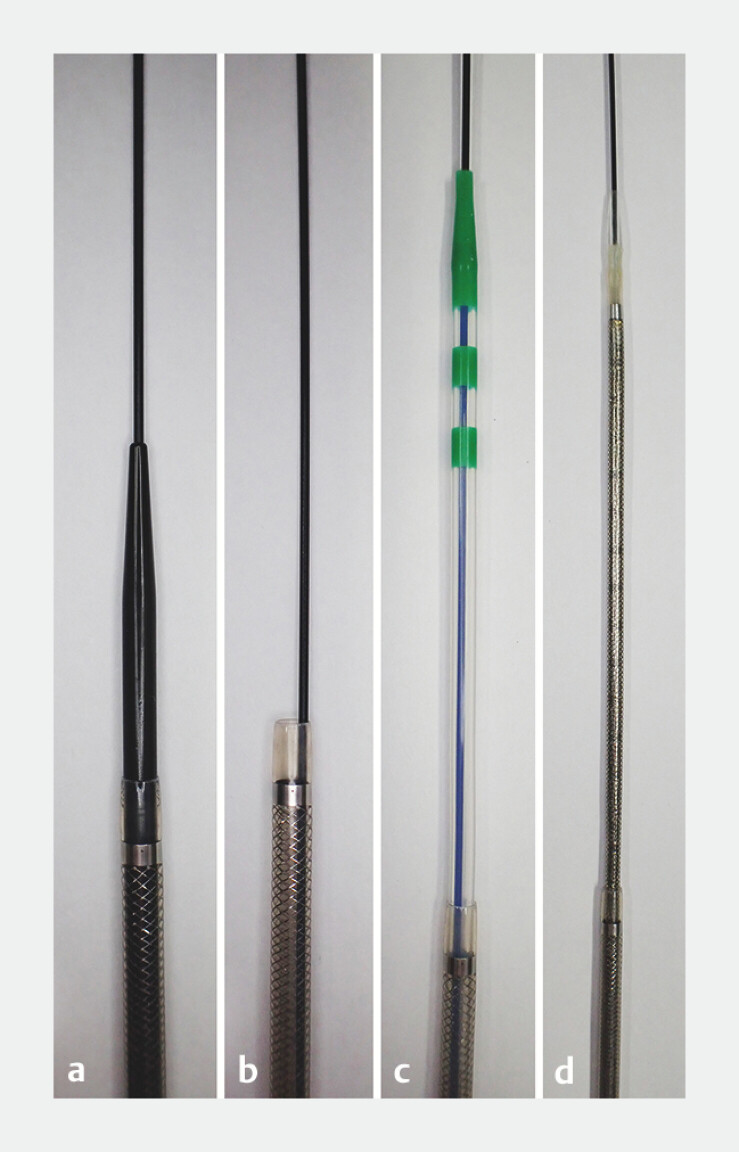
**a** A guiding sheath (EndoSheather; Piolax Medical Devices, Kanagawa, Japan) is 1707 mm in length and consists of a 7.2F outer catheter and an inner catheter with a highly tapered tip. **b** When the inner catheter is removed, devices with an outer diameter ≤ 6F can be passed through the outer catheter. **c** In the present case, a standard catheter with a maximum outer diameter of 5.5F (PR-V435Q; Olympus Medical Systems, Tokyo, Japan) **d** and an uncovered metal stent with a 5.4F delivery system (ZeoStent V; Zeon Medical, Tokyo, Japan) were used.

**Fig. 2:**
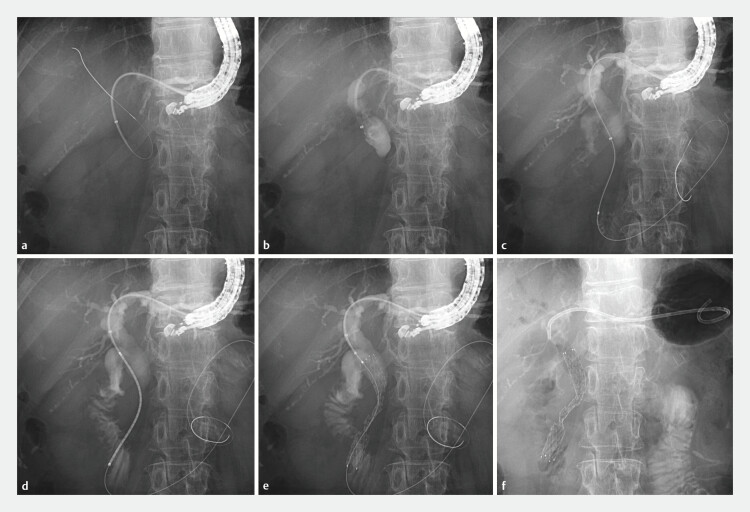
**a** The left intrahepatic bile duct was punctured from the stomach using a 19G needle, and a 0.025-inch guidewire was advanced into the common bile duct. Subsequently, the guiding sheath was introduced into the common bile duct. **b** The inner catheter and guidewire were then removed, followed by sufficient bile aspiration and contrast medium injection. **c** Next, a 5.5F standard catheter was inserted through the outer catheter of the guiding sheath, allowing the guidewire to be advanced across the stricture into the duodenum. **d** Thereafter, a 5.4F diameter delivery system for a metal stent (10 × 80 mm) was inserted through the guiding sheath, with additional pushability support provided by the sheath, **e** and the stent was deployed antegrade across the stricture. **f** Finally, a 7F single-pigtail plastic stent was placed for hepaticogastrostomy to maintain the access route.

Endoscopic ultrasound-guided antegrade stenting with guiding sheath assistance in a patient with malignant distal biliary obstruction.Video 1

This technique has the potential to enhance safety and simplicity of EUS-AGS by reducing bile leakage during the procedure and providing backup-support during device insertion.

## References

[1] Ishiwatari H Ogura T Hijioka S EUS-guided hepaticogastrostomy versus EUS-guided hepaticogastrostomy with antegrade stent placement in patients with unresectable malignant distal biliary obstruction: a propensity score-matched case-control study Gastrointest Endosc 2024 100 66 75 38382887 10.1016/j.gie.2024.02.012

[2] Itonaga M Ashida R Hatamaru K Endoscopic ultrasound-guided hepaticogastrostomy vs. antegrade metal stent placement keeping an access route in patients with malignant biliary obstruction Int J Clin Oncol 2024 29 1500 1508 38972023 10.1007/s10147-024-02584-2

[3] Kato A Yoshida M Hori Y The novel technique of drainage stenting using a tapered sheath dilator in endoscopic ultrasound-guided biliary drainage DEN Open 2023 4 e303 10.1002/deo2.303 37873053 PMC10590603

